# Analyzing nonverbal listener responses using parallel recordings of multiple listeners

**DOI:** 10.1007/s10339-012-0434-3

**Published:** 2012-02-19

**Authors:** Iwan de Kok, Dirk Heylen

**Affiliations:** Human Media Interaction Group, University of Twente, Enschede, The Netherlands

**Keywords:** Corpus analysis, Listener responses, Social signals

## Abstract

In this paper, we study nonverbal listener responses on a corpus with multiple parallel recorded listeners. These listeners were meant to believe that they were the sole listener, while in fact there were three persons listening to the same speaker. The speaker could only see one of the listeners. We analyze the impact of the particular setup of the corpus on the behavior and perception of the two types of listeners: the listeners that could be seen by the speaker and the listeners that could not be seen. Furthermore, we compare the nonverbal listening behaviors of these three listeners to each other with regard to timing and form. We correlate these behaviors with behaviors of the speaker, like pauses and whether the speaker is looking at the listeners or not.

## Introduction

In a conversation, participants display when they are listening various behaviors in response to the contributions to the conversation of the speaker. These take the form of nonverbal behaviors such as head nods and shakes, various kinds of facial expressions or vocalisations such as uh-huh, hmm, etcetera. These so-called listener responses (Bavelas et al. 2000; Dittmann and Llewellyn 1968), including what are commonly known as backchannels (Yngve 1970), are intimately connected with the contributions of the speaker. They signal that the contribution is being attended to, understood, agreed upon or some other attitudinal or affective reaction to it (Allwood et al. 1992; Bavelas et al. 2000; Clark 1996). This dependence of the occurrence of a listener response on the contribution of the speaker has prompted many studies on the characteristics of the speaker’s contribution that might act as cues or triggers for the responses both from a linguistic perspective (Dittmann and Llewellyn 1968) and from a computational perspective. The hope is to find algorithms that can produce appropriate responses in spoken dialogue systems or embodied conversational agents based on features derived from the speaker’s contribution (Heylen et al. 2011).

The assumption behind these studies is that listener responses do not occur randomly, or at the listeners’ whims, but that there is some kind of dependence on the speaker’s contribution. This seems rather intuitive. If, for instance, the function of a response is to signal understanding or agreement, it makes sense that it should occur near the place where a speaker is completing or has just completed an informative unit. Hence, the importance of the “phonemic clause” unit is argued in Dittmann and Llewellyn’s work (1967). Similarly, a speaker’s need for grounding—finding out whether listeners are attending and understanding what they are saying—may be marked by certain cues (gaze, intonation, etc.) that invite a response. But although there may be such a dependency between a speaker’s contribution and a listener’s response, there is no strict, mechanical rule here. A listener may ignore the speaker’s invitation and the speaker may just as well continue taking the absence of a signal of misunderstanding as a positive signal of grounding. In other cases, the enthusiasm of listeners about the speaker’s contribution may prompt them to nod throughout. The computational modeling of listener behaviors can thus only partly rely on the cues from the speaker’s contribution but needs to take into account also models of emotion or personality and many other factors.

In order to understand more about the factors influencing the production of listener’s responses, the type of dependence on characteristics of the speaker’s contribution and the variation between listeners, we have collected a special kind of corpus in which we recorded multiple listeners interacting in parallel with the same speaker.

In this paper, we will present this corpus in short and provide some analyses of the listening responses that can be observed in the corpus. We address two main questions. The first relates to checking the validity of the naturalness of the behaviors that we recorded given our particular construction of the corpus. The second question concerns an investigation into the notion of “response opportunity” or the notion of dependence of the response on characteristics of the speaker’s contribution.

We will first introduce the corpus on which these analyses have been performed. Then, we will discuss the definitions of what we call listener responses and response opportunities. This is followed by a study to validate that we recorded natural behavior using this particular construction of the corpus. The relation between the speaker’s behavior and the responses of the listener is studied after that and finally we will draw our conclusions.

## The MultiLis corpus

The MultiLis corpus (de Kok and Heylen 2011) is a Dutch spoken multimodal corpus of 32 mediated face-to-face interactions totaling 131 min. Participants (29 male, 3 female, mean age 25) were assigned the role of either speaker or listener during an interaction. In each session, four participants were invited to record four interactions. Each participant was once speaker and three times listener.

**Fig. 1:**
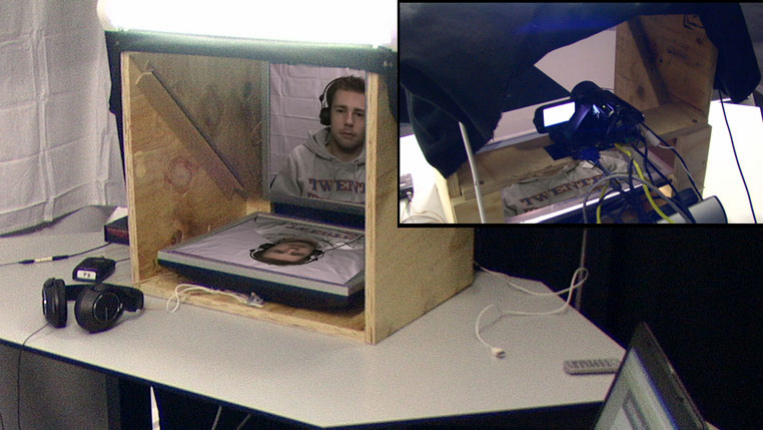
*Picture* of the *cubicle* in which the participants were seated. It illustrates the interrogation mirror (transparent from one side) and the placement of the camera behind it, which ensures eye contact

What is unique about this corpus is the fact that it contains parallel recordings of three individual listeners in interaction with the same speaker, while each of the listeners was tricked into believing to be the sole listener. The speakers saw only one of the listeners, believing that they had a one-to-one conversation. We will refer to this listener, who can be seen by the speaker, as the *displayed listener*. The other two listeners, who could not be seen by the speaker in the interaction, will be referred to as *concealed listeners*. All listeners were placed in a cubicle and saw the speaker on the screen in front of them, illustrated in Fig. 1. The camera was placed behind an interrogation mirror (transparent from one side), positioned directly behind the position on which the interlocutor was projected. This made it possible to create the illusion of eye contact.

To ensure that the illusion of a one-to-one conversation was not broken, interaction between participants was limited. Speakers and listeners were instructed not to ask for clarifications or to elicit explicit feedback from each other, so no turn-switching would take place. The speaker received a task of either watching a short video clip before the interaction and summarizing it to the listener or learning a recipe in the 10 min before the interaction and reciting it to the listener. The listener needed to remember as many details of what the speaker told as possible, since questions about the content were asked afterward.

Besides the questions on the content of the interaction, they also filled in a questionnaire about the interaction itself measuring the rapport between the interlocutors. After the whole session, participants were presented a questionnaire about the setup of the experiment.

In the corpus, annotations are available of head gestures (different type of nods, shakes, turns, tilts), eye gaze (looking at the listener or not), mouth gestures (smiles, lip press), and eye brows (frown, raised). More details on the annotations can be found in de Kok and Heylen (2011). Furthermore, we extracted pauses (segments of silence of at least 100 ms) using the Dutch automatic speech recognizing software SHoUT (Huijbregts 2008).

## Listener responses and response opportunities

As we pointed out in the introduction, there is no hard-and-fast requirement on a listener to produce a response at a given time. Listener responses are optional. Even though listener responses are important for a successful completion of an interaction, it does not mean that when a listener will not provide a response at a certain moment the conversation will immediately break down. It is known that individuals differ in their choice in timing and type of listener responses, but we have no real understanding yet of the causes of these differences.

The MultiLis corpus allows us to look into these causes. The corpus offers three listeners who react to the exact same context. In the corpus, the onset of each listener response is annotated. The moments where at least one listener produces a response can be called *response opportunities.* If there had been more listeners in each interaction, then we would probably identify more response opportunities. So, even though the corpus will not provide us a complete coverage of all the possible response opportunities in the interaction (there are also response opportunities where none of the listeners responded), the coverage is a lot wider than when we only have one listener.

For each response opportunity, we annotated how many listeners have provided a listener response [for details on how this was done, see (de Kok et al. 2010)]. In the corpus, there are 1,125 response opportunities with 1 of the listeners producing a response, 462 response opportunities with 2 listeners providing responses and 128 with 3 listeners responding at the same time. There thus seems to be a graded optionality in the response opportunities, by the fact that in some opportunities all three listeners respond, and in others only a subset of the listeners. The question now is what makes these opportunities more compelling to response? Do speakers produce different, or more cues inviting a response at these moments than at the other moments? And do listeners act in the same way when they respond at the same time? These are some of the questions we will investigate in the remainder of this paper. Before we turn to these questions, though, there is another question we would like to address first and this is whether there are significant differences in the behaviors between the displayed and the concealed listeners.

## Are all three listeners equal?

Listeners are not inactive bystanders in an interaction, but are involved in the conversation as much as the speaker (Bavelas et al. 2000). Listeners respond to speakers but speakers equally respond and adapt to the contributions to the conversation of the listeners. By disconnecting the feedback loop from the listener to the speaker for the two concealed listeners, the setup may have influenced the behavior and/or the perceived experience of these listeners. In this study, we aim to investigate whether this is the case.

We do this in three ways. We will first analyze the subjective measures taken after each interaction to see whether the listeners have noticed this during the interaction. Next, we will analyze the behavior the listeners have displayed. Finally, we will perform a perceptive study to see whether observers can detect the differences (if any) in the behavior between displayed and concealed listeners. One should keep in mind that the setup of the experiment ensured that same persons are in each of the groups; data from each person are included once in the displayed listener group and twice in the concealed listener group, but each time from a different interaction.

### Subjective measures

After each interaction, the listeners filled out a questionnaire measuring the rapport between the listener and the speaker. The measure consists of 10 5-point Likert scale questions and is an adapted version of the rapport measure used by Gratch et al. (2006) with additional questions from the Inventory of Conversation Satisfaction scale from White (1989). Some sample questions are “I was able to motivate the speaker to tell his story well” and “The speaker paid attention to me”.

The displayed listeners reported a significantly higher rapport rating for the interactions than the concealed listeners (3.39 vs 3.05, respectively, *p* = 0.014). This is inline with what one would expect, as the speaker does not respond to concealed listeners.

After all four interactions, we explained the setup of the experiment, and we asked whether the participants had become aware of the fact that the speaker could not see them in two out of three situations in which they were the listener. Of the 31 participants that completed this questionnaire, 14 claimed they noticed this manipulation, but only 6 of them could identify the correct interaction in which they were the displayed listener, 5 guessed a wrong interaction and 3 reported to have no idea. So even though they noticed, only about half of them were able to identify the correct interaction. Of the 17 participants that did not notice, 5 participants guessed the correct interaction in which they were the displayed listener, 11 made the wrong guess, and 1 reported to have no idea.

### Objective data

Another way to look at the differences is to see whether there are any differences in the behaviors that were actually displayed by the two types of listeners. Looking at the amount of responses per minute, the displayed listeners gave 7.7 responses per minute and the concealed listeners gave 6.8 responses per minute. This is on average 12% less responses from the concealed listeners opposed to displayed listener. However, this difference is not statistically significant, due to the big variance between participants (*p* = 0.33).

If the concealed listener would (consciously or unconsciously) notice that they are not seen by the speaker, they would do this as the interaction progresses. At the beginning, they will not know that they are concealed; they may only notice that the speaker does not react to their response later on and give fewer responses as a result of that. If this is true, we expect the difference in response rate between displayed listeners and concealed listeners to increase over time.

**Fig. 2:**
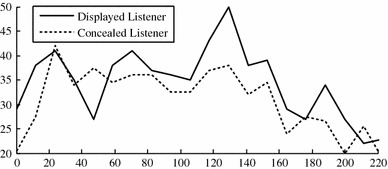
Responses over time from the displayed listener (*continuous*) and the concealed listeners (*dotted*)

To test whether this is the case or not, we have plotted the number of responses over time for the displayed listeners (continuous) and the concealed listeners (dotted) in Fig. 2. We only used the 15 interactions which lasted longer than 4 min. We divided the first 4 min into 20 windows and counted how many responses the listeners gave within that time frame. It shows that the number of responses from the concealed listeners is usually smaller than from the displayed listeners, but we do not see that the gap between the two lines increases over time.

### Perceptive study

So, the objective data have not given us significant results which discriminate the behavior of the displayed listener from the concealed listeners. There are indications that concealed listeners give fewer responses, but these results are not significant due to the large variance between listeners. Possibly, the changes in behavior are more subtle than one can detect by analyzing objective data. Humans are very capable of noticing subtle changes in behavior. It may only be one precisely timed head nod which discriminates the displayed listener from concealed listeners. This one head nod will get lost in the numbers of an objective analysis, but humans are highly susceptible to such nonverbal cues. Therefore, we have performed a perceptive study where we asked observers to look at the interaction between the speaker and the three listeners, with the task to point out the displayed listener.

We invited 16 participants, recruited at the faculty, to participate in the study. The corpus was split into 48 segments ranging from 1:45 to 3:40 min. Each participant was shown, through a webpage, 6 segments of the corpus. In the segments, the speaker was presented in the top left corner. The three listeners were positioned in the other three corners of the screen. In which corner the displayed listener was placed was varied. They could pause and repeat the whole or part of the segment. For each of the segments, they were asked to identify the displayed listener among the three listeners. Every segment was presented to two participants.

In total, the participants answered correctly in 43 of 96 segments (45%), which is significantly better than chance [*P*(*X* > = 43)] = 0.01, with an a priori chance of 33%. The amount of correct answers varied from 0 to 6. There were 8 segments where both participants identified the correct listener, 27 where one participant identified the correct listener and 13 where none of the participants identified the correct listener. Informal interviews with the four participants who identified the correct displayed listener at least 4 out of 6 times revealed that their strategy was to look for reactions of the speaker to one of the listeners. The listener to which the speaker reacted was chosen to be the displayed listener. They especially paid attention to the timing of smiles. They looked for moments where a speaker reacts to a smile by one of the listeners (by smiling or any other type of reaction). The listener who the speaker reacted to must have been the displayed listener.

## Response opportunities and speaker behavior

As we mentioned in the introduction, several studies have investigated how the placement of listener responses may be related to behavior of the speaker’s turn. In this section, we look at the notion of graded optionality of response opportunities and how this correlates with behaviors of the speakers. Are there cues the speaker provides, like pausing and/or looking at the listener, that encourage listeners to provide a response at a certain moment? To do this, we will compare the speaker’s behavior at response opportunities to which 1, 2, and 3 listeners responded to each other. We will also look into agreement in the type of gestures the listeners have performed at these response opportunities.

To ensure we can regard the responses of the displayed and the concealed listeners as equal, we will check before each analysis, whether the disruption of the closed interaction loop for the concealed listener has resulted in a difference in behavior on the specific aspect we analyze. We do this by comparing the displayed and concealed listener responses to each other.

### The relation between pauses and responses

**Fig. 3:**
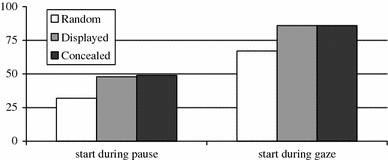
Shows the percentage of responses from either displayed or concealed listeners which start while the speaker is pausing or looking at the listener

The first feature we looked at is pauses. The left bar graph in Fig. 3 shows the percentage of responses from either the displayed (gray) or concealed listener (black) which start during a silence (or pause) from the speaker (both a little under 50%). There is no significant difference between the two types of listeners, so the disruption of the closed interaction loop had no impact on the timing of their responses with regard to pause.

**Fig. 4:**
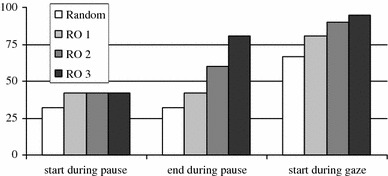
Shows the percentage of response opportunities (RO), grouped by the number of responses in the response opportunity, for which the start is during a pause, end is during a pause or the start is while the speaker is looking at the listener. Random is the percentage of pause or “gaze at the listener” that is totally present in the data

In the left bar graph in Fig. 4, one can see the percentage of response opportunities which start during a pause. If one would randomly distribute the start times during the interaction, 32% would be during pauses, since on average speakers pause 32% of the time. There are no differences between response opportunities with one, two, or three responses. Around 42% of all response opportunities start during a pause, which is significantly above random, χ^2^(1, *N* = 3,470) = 39.0, *p* < 0.01, but there is no significant difference between response opportunities with a different number of responses, χ^2^(1, *N* = 1,735) = 0.05, *p* < 0.97.

Interestingly, when one looks at the end times of the response opportunities, one can see a big difference (center bar graph in Fig. 4). Again all response opportunities occur significantly more often during pause than random distribution would predict, χ^2^(1, *N* = 3,470) = 113.74, *p* < 0.01. For response opportunities with one response, the percentage remains 42%, since start and end times are the same for those, but end times of response opportunities of two responses are 60% of the time during a pause of the speaker. For response opportunities of three responses this even increases to 81%. Between response opportunity groups, the results are also significant, χ^2^(1, *N* = 1,735) = 113.74, *p* < 0.01.

This means that especially response opportunities with three responses are situated around the end of an utterance which is followed by a pause. Whereas some listeners place their listener responses during the end of the utterance, others place them in the pause which follows. Thus, the window of opportunity to provide a backchannel starts during the end of the utterance and continues during the pause. In the 166 cases where only the end is during a pause (response opportunities with two and three responses combined), the mean overlap between the response opportunity and the utterance is 217 ms.

To go into this a little more deeply, we looked at the distance in ms from the start of a response and the closest start time of a pause. If the start of the response is before the start of a pause, a negative distance is recorded.

**Fig. 5:**
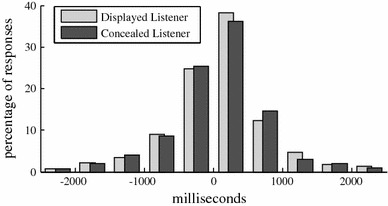
Distribution of the timing of responses in relation to the start of the nearest pause for displayed listeners and concealed listeners. The timing distribution is similar for both listener types

The mean location of the 2,433 responses in this corpus is 81 ms after the start of a pause, with a standard deviation of 809 ms. A random distribution of responses would result in a mean of 23 ms before the start of a pause, with a standard deviation of 956 ms. In Fig. 5, the distributions of the timing of responses in relation to the start of the nearest pause is presented for both displayed listeners and concealed listeners. A pair-sampled t-test shows there is no significant difference (*p* = 0.19) between the two groups of listeners.

**Fig. 6:**
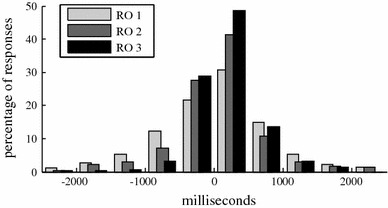
Combined *histogram* of the distance of each response to the closest silence, where negative distances are responses which are given before the closest silence. The responses at RO with responses by three listeners (RO3) are situated less before the closest silence than responses part of RO with one (RO1) or two responses (RO2)

If we compare the individual responses in response opportunities with one response to the individual responses in response opportunities with two or three responses, we can see that the more listeners reacted at the same time, the later the responses are with regard to the start of a pause (46 ms after a pause for one response, 91 ms for two responses and 162 ms for three responses) and also the standard deviation decreases (928, 750 and 477 ms, respectively). The histogram distributions are plotted in Fig. 6. Responses of response opportunities with three responses are only rarely situated more that 1 s before the closest pause. Furthermore, the percentage of responses which is a within 500 ms after the start of a pause is a lot higher. This further acknowledges the relation between pause and the timing of a response.

The pause itself is probably not the cue to which the listeners respond. It is more likely that the close of a grammatical clause is the cue, as mentioned by Dittman and Llewllyn (1967). Since the close of a grammatical clause is likely to be followed by a pause, the relation between pause and responses is found. This also explains why quite a few responses are located just before the start of a pause, instead of after. In interaction, interlocutors usually predict the ending of a sentence or turn, to plan their response and often respond (partly) based on that prediction, before their interlocutor has completed their sentence (Sacks et al. 1974).

### The relation between gaze and responses

The second feature we looked at is gaze. The right bar graph in Fig. 3 shows the percentage of responses from either the displayed (gray) or concealed listener (black) which start during a period in which the speaker was looking at the listener (both around 85% of the time). There is no significant difference between the two types of listeners, so the disruption of the closed interaction loop had no impact on the timing of their responses with regard to gaze.

In the right bar graph in Fig. 4, one can see the percentage of response opportunities which start while the speaker is looking at the listener. In our corpus, the speaker looks on average 67% of the time at the listener, so randomly distributed responses would start 67% of the time when the speaker is looking at the listener. Comparing all response opportunities to the random condition, we find that speakers look significantly more at the listener than random distribution would predict, χ^2^(1, *N* = 3,470) = 139.94, *p* < 0.01. For response opportunities with one response, the percentage of response opportunities which start while the speaker is looking at the listener is 81%. For response opportunities with two responses, this percentage increases to 90% and for response opportunities with three responses even to 95%. These increases are significant, χ^2^(1, *N* = 1,735) = 30.6, *p* < 0.01. For gaze during end, the percentages are a little lower for response opportunities with two responses (86%) and for response opportunities with three responses (94%).

These results support previous findings that looking at the listener as a speaker is a cue for a listener to respond (Bavelas et al. 2002; Heylen 2006; Kendon 1967). More interestingly, we can see that this cue is more dominantly present during response opportunities of three listener responses than during response opportunities of one or two responses.

### Agreement in head gesture type between listeners

We have seen that the speaker behaviors have an influence on the timing of listener responses, but do they also have an influence on the selection of the gesture which is performed by the listener? If two or three listeners respond at the same *time*, do they also respond in the same *way*? To answer this, we look at the response opportunities where at least two listeners responded and look at agreement in head gesture type.

The main head gesture types available in the corpus are *Nod* (characterized by a downward stroke), *Backnod* (characterized by an upward stroke), and *Double nod* (two nods in quick succession with the same amplitude). Of each of these head gesture type, there is a *Lingering* variant. These head gestures continue for a period of time in decreasing amplitude. If the amplitude is increased, a new head gesture is annotated. The corpus also included the labels *Downstroke* and *Upstroke*, which are single up or down movements. For the next analyses, these are considered as *Nod* and *Backnod*, respectively. All the other labels are combined in the label *Other*.

For the first analyses, we looked whether the three listeners used the same head gesture when they reacted at the same time. For each response opportunity with at least two responses, we noted the head gestures type the listener used in their response. This was Nod, Backnod, Double Nod, or Other. Then, we calculated Krippendorff’s α coefficient (Hayes and Krippendorff 2007) to measure the agreement between the two or three listeners, resulting in an α of 0.082. So no significant agreement is found in the head gesture type the listeners used.

We also looked at whether there was agreement between listeners in their use of the lingering head gestures. Each head gesture was coded as either Lingering or Not Lingering. Again Krippendorff’s α coefficient was calculated to measure agreement and no agreement is found (α = 0.017).

So, since there is no agreement between listeners in the head gesture type selection, this selection does not seem to be determined by the context of the response opportunities in which the response is placed, but rather by internal motivators. Which internal motivator (for instance, personality, mood, personal preference) is the key factor in this choice is yet to be determined.

## Conclusion

In this paper, we have studied nonverbal listener responses on a corpus with multiple parallel recorded listeners. To check whether the particular construction of the corpus had an influence on the behavior of the concealed listeners, we have looked into the differences between displayed and concealed listeners. We have found that displayed listener reported statistically significant higher rapport than concealed listeners. The objective data, however, shows no statistically significant decrease in amount of responses for concealed listeners. In the perceptive study, some observers are able to discriminate the displayed listeners from the other two listeners by paying close attention to the timing of individual behaviors of the listeners (especially smiles) and the reactions of the speaker to these behaviors, while most observers are unable to discriminate them.

When we look at the speaker behaviors near response opportunities, we have seen that response opportunities with responses from three listeners are situated around the end of a grammatical clause more often than response opportunities with a response from one or two listeners. Furthermore, they are more often while the speaker is looking at the listener. Analysis of the form of the listener response of different listeners to the same response opportunity showed that there is no agreement between the displayed behavior. This suggests that the form of the listener response is not directly dictated by the speaker’s behavior and context, but more by the listener’s characteristics. More research is needed to understand which factors influence the form of listener responses as the studies presented in this paper are inconclusive.

## References

[CR1] Allwood J, Nivre J, Ahlsén E (1992). On the semantics and pragmatics of linguistic feedback. J Semant.

[CR2] Bavelas JB, Coates L, Johnson T (2000). Listeners as co-narrators. J Pers Soc Psychol.

[CR3] Bavelas JB, Coates L, Johnson T (2002). Listener responses as a collaborative process: the role of gaze. J Commun.

[CR4] Clark HH (1996) Using language. Cambridge University Press, Cambridge

[CR5] de Kok I, Heylen D, Esposito A, Esposito A, Martone R (2011). The MultiLis corpus—dealing with individual differences of nonverbal listening behavior. Toward autonomous, adaptive, and context-aware multimodal interfaces: theoretical and practical issues.

[CR6] de Kok I, Ozkan D, Heylen D, Morency L-P (2010) Learning and evaluating response prediction models using parallel listener consensus. In: Proceedings of the ICMI–MLMI ‘10 international conference on multimodal interfaces and the workshop on machine learning for multimodal interaction

[CR7] Dittmann AT, Llewellyn LG (1967). The phonemic clause as a unit of speech decoding. J Pers Soc Psychol.

[CR8] Dittmann AT, Llewellyn LG (1968). Relationship between vocalizations and head nods as listener responses. J Pers Soc Psychol.

[CR9] Gratch J, Okhmatovskaia A, Lamothe F (2006). Virtual rapport. Proc Intell Virtual Agents.

[CR10] Hayes AF, Krippendorff K (2007). Answering the call for a standard reliability measure for coding data. Commun Methods Meas.

[CR11] Heylen D (2006). Head gestures, gaze and the principles of conversational structure. Int J Hum Robot.

[CR12] Heylen D, Bevacqua E, Poggi I, Cowie R, Pelachaud C, Petta P (2011). Generating listening behavior. Emotion-oriented systems. The humaine handbook.

[CR13] Huijbregts M (2008). Segmentation, diarization and speech transcription: surprise data unraveled.

[CR14] Kendon A (1967). Some functions of gaze direction in social interaction. Acta Psychol.

[CR15] Sacks H, Schegloff EA, Jefferson G (1974). A simplest systematics for the organization of turn-taking for conversation. Language.

[CR16] White S (1989). Backchannels across cultures: a study of Americans and Japanese. Lang Soc.

[CR17] Yngve VH (1970) On getting a word in edgewise. In: Sixth regional meeting of the Chicago linguistic society, pp 657–677

